# Harnessing the Bio-Instructive Placental Extracellular Matrix: Structural Properties, Decellularization, and Applications in Regenerative Medicine

**DOI:** 10.3390/ijms27167259

**Published:** 2026-08-14

**Authors:** Gianluca Fontana, Giulio Innamorati, Luca Giacomello

**Affiliations:** Department of Surgical Sciences, Dentistry, Gynecology and Pediatrics, University of Verona, 37134 Verona, Italy; gianluca.fontana@univr.it (G.F.); giulio.innamorati@univr.it (G.I.)

**Keywords:** placenta, biomaterials, decellularization, extracellular matrix, growth factors, 3D printing

## Abstract

The persistent shortage of donor organs and the inherent drawbacks of autologous grafts highlight the urgent need for advanced biomaterial scaffolds in regenerative medicine. Synthetic polymers and animal-derived matrices offer structural support, yet they frequently lack the biological complexity of native tissue or carry translational liabilities—xenogeneic antigens, pathogen transmission risk, and batch variability. This review positions the human placenta as a compelling, ethically sourced, and abundant reservoir for fully human, xeno-free biomaterials. We examine the placenta’s distinct anatomical compartments and their rich complement of extracellular matrix (ECM) proteins, growth factors, and bioactive cytokines. These components confer potent pro-angiogenic, anti-inflammatory, antimicrobial, and immunomodulatory properties, enabling precise direction of cellular behavior and tissue regeneration. We systematically assess recent advances in decellularization and the processing strategies required to preserve these bioactivities while eliminating immunogenic material. By integrating current tissue engineering applications with the regulatory and ethical frameworks shaping clinical translation, we argue that placenta-derived matrices are uniquely positioned to transcend the limitations of conventional scaffolds and serve as a robust platform for future regenerative therapies.

## 1. Introduction

Autologous grafts for replacing damaged tissue are inherently invasive, limited by the availability of healthy harvest sites, and carry a significant risk of morbidity [1]. Alternatively, allogeneic transplantation from donor organs usually requires lifelong immunosuppression to prevent rejection, increasing the risk of serious infections and other complications [2]. Prolonged immunosuppression, together with the chronic shortage of donor organs—which leads to long transplant waiting lists and high patient mortality [3]—highlights the critical need for advanced biomaterial scaffolds that can direct cell behavior and support targeted tissue regeneration.

To build functional replacement tissues, early regenerative medicine approaches used synthetic polymer-based scaffolding systems (for example, polylactic acid or polycaprolactone). These frameworks were designed as temporary templates that provide necessary structural support —to cells seeded in vitro, before implantation, or recruited in vivo from the host [4]. However, while these materials offer robust structural integrity, their bio-inert nature often results in poor cell adhesion and suboptimal tissue integration unless functionalized with bioactive additives or ceramics [4]. Therefore, an ideal scaffold must provide structural support while directing new tissue formation. This has triggered strong interest in bio-instructive materials that better mimic the native tissue microenvironment and provide cues for cellular adhesion, proliferation, and differentiation [5].

Biological scaffolds derived from decellularized animal tissues—such as bovine collagen or murine tumor-derived matrices like Matrigel^®^—represent a step forward in this direction, but introduce their own translational bottlenecks: residual xenogeneic antigens, risk of zoonotic pathogen transmission, and significant batch-to-batch variability [6]. Fully human, xeno-free alternatives that combine structural fidelity with biological complexity, while bypassing these limitations, remain an unmet need in the field.

Among the most promising candidates for a fully human, xeno-free bio-instructive scaffold is the placenta. Obtained as a natural by-product of childbirth and normally discarded as medical waste, it represents an ethically sourced and abundantly available biological resource [7]. Its therapeutic utility has been documented for over a century: Davis’s 1910 application in skin grafting [8] marked the beginning of a clinical trajectory that today spans multiple medical specialties and a multi-billion dollar biologics market [9]. These clinical successes were enabled by the placenta’s unique biological composition—including pro-angiogenic, anti-inflammatory, anti-microbial, and immunomodulatory properties detailed in the following sections. Nevertheless, significant barriers to widespread clinical translation remain, including the absence of standardized processing and manufacturing protocols, regulatory uncertainty, and complex ethical considerations arising from the genetic link between placental tissue and the offspring.

In this review, we discuss recent advancements in placenta-based biomaterials, covering their structural biology and cellular components, processing and decellularization strategies, applications in tissue engineering and 3D bioprinting, and the regulatory and ethical landscape governing their clinical translation. Particular attention is provided to the emerging imperative for xeno-free, fully human biofabrication platforms.

## 2. The Placenta as a Biologics Reservoir

The placenta is a valuable therapeutic source because of its unique biological make-up. The placenta’s ECM is made up of structural proteins, such as collagen and glycosaminoglycans (GAGs), as well as a diverse array of bioactive cytokines and growth factors that include: vascular endothelial growth factor (VEGF), hepatocyte growth factor (HGF), platelet-derived growth factor (PDGF), transforming growth factor-beta (TGF-β), epidermal growth factor (EGF) and basic fibroblast growth factor (bFGF). All of these factors work together to enhance tissue repair by stimulating re-epithelialization, enhancing neo-vessel formation (through angiogenesis), reducing fibrosis, and suppressing inflammation in injured or damaged tissue [8]. Regardless of their specific anatomical compartment of origin, placental biomaterials share several fundamental therapeutic properties. They have shown angiogenic activity due to a rich reservoir of endogenous mediators that stimulate the rapid neovascularization critical for graft integration [10]. Moreover, these factors exert anti-inflammatory and anti-fibrotic effects that prevent excessive scarring during wound healing [11]. Specific compartments, such as the amniotic membrane (AM) and chorion, add broad-spectrum antimicrobial protection. Finally, the native cellular compartments of the placenta exhibit inherently low classical Major Histocompatibility Complex (MHC) antigen expression [12]. This inherent immune privilege not only facilitates the allogeneic use of cellularized placental grafts (such as Mesenchymal Stromal Cells (MSCs) and intact membranes) without aggressive immunosuppression, but it also ensures that decellularized extracellular matrix (dECM) derived from these tissues has a generally low baseline immunogenicity, mitigating the risk of rejection even if trace cellular fragments remain after processing. Although these features are present across all placental compartments, their expression levels and structural contexts vary significantly. This has important implications regarding each compartment’s suitability in different regenerative medicine applications (Table 1).

### 2.1. Structural Compartments of the Placenta

**Figure 1:**
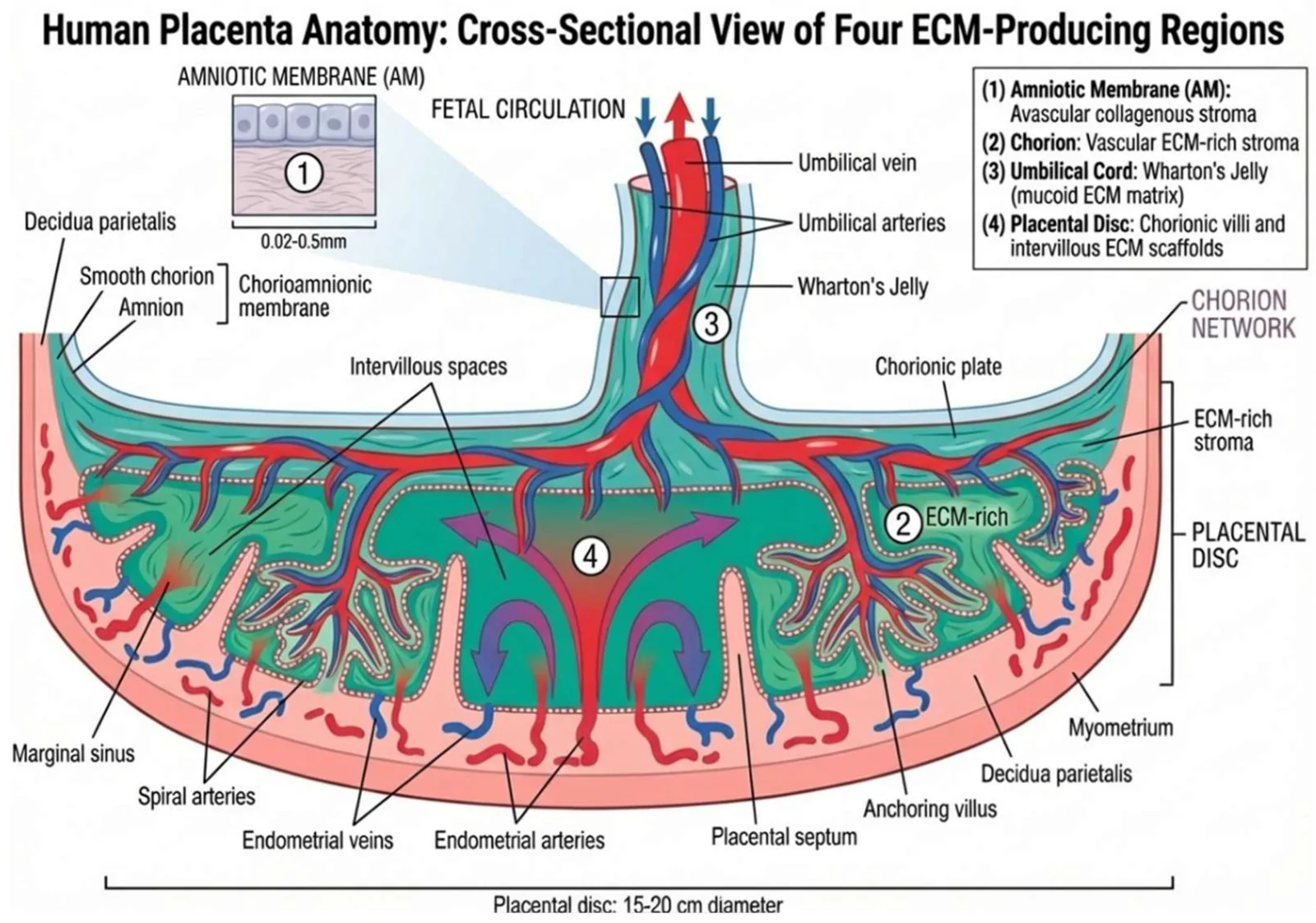
Cross-sectional anatomy of the human placenta highlighting the four primary ECM reservoirs utilized for biomaterials: (1) the avascular amniotic membrane (AM), (2) the vascularized chorion, (3) the umbilical cord and Wharton’s Jelly, and (4) the placental disc. Each compartment provides unique structural and bioactive properties for regenerative applications. Image generated by the authors.

The placental ECM can be extracted from four separate areas (Figure 1): the AM; chorion (chorionic membrane); umbilical cord, including Wharton’s jelly (WJ); and the placental disc (consisting of chorionic plate and cotyledons).

The AM is a thin membrane with no blood vessels which is about 0.02–0.5 mm in thickness [13], where the exchange of metabolites and oxygen occurs by passive diffusion [14]. Lacking vascularization, AM ECM represents an ideal source for the reconstruction of avascular tissues (i.e., cornea and cartilage) in which neovascularization would not be desirable. There are three distinct layers found within the AM: (i) a single layer of epithelial cells that secrete growth factors (HGF, EGF, bFGF, TGF-β1, TGF-β2) [15]; (ii) a basement membrane composed of adhesive proteins such as laminin and collagen type IV [16]; and (iii) a supportive stroma made up of mostly interstitial collagen type I and type III [14]. The modern clinical adoption of the AM began with its success as an intact biological dressing in ophthalmology, specifically for corneal repair and ocular surface reconstruction [17]. In its native, cellularized state, the AM leverages its epithelial and stromal layers to provide anti-inflammatory, anti-fibrotic, and antimicrobial effects that actively promote epithelial regeneration. Today, however, its applications are distinctly categorized based on tissue processing. Minimally manipulated, cellularized AM continues to be widely used for ocular surface reconstruction and clinical wound care. Conversely, decellularized AM (dAM)—which is stripped of cells but retains the structural and biochemical cues of the basement membrane and stroma—is increasingly utilized as a biologically active, low-immunogenicity scaffold for complex tissue engineering applications [18].

The chorion is a vascularized tissue compared to the avascular AM and expresses a broader angiogenic growth factor profile. These include Adiponectin, Angiogenin (ANG), ANG-2, bFGF, endothelium-specific growth factor, HGF, insulin-like growth factor-1 (IGF-1), platelet-derived growth factor AA (PDGF-AA) and BB (PDGF-BB) isoforms, and two forms of tissue inhibitors of metalloproteinases (TIMP)-2 and -4 [19]. This profile of growth factors makes it an ideal candidate for use in tissue engineering applications requiring the rapid formation of new blood vessels such as vascular grafts and engineered constructs with high metabolic demand [20].

The umbilical cord joins the fetus to the placenta through one vein and two arteries that are enveloped in a thick, gelatinous environment known as WJ [21]. The ECM of the WJ is formed from proteoglycans, hyaluronic acid and a honeycomb-like structure of collagen (Types I, III, V, and VI). The ECM protects the blood vessels from being compressed by surrounding tissues throughout gestation and gives the umbilical cord unique properties of viscoelasticity and hydration [22]. For this reason, WJ could be a potential source of ECM for creating hydrogels that can provide lubrication, cushioning and filling space, such as for cartilage tissue engineering and joint repair.

The placental disc is the primary structural compartment of the placenta (measuring approximately 15–20 cm in diameter at full term), serving as the main site for the exchange of oxygen, carbon dioxide, and nutrients between mother and fetus [23]. It has the maternal side basal plate and the fetal side chorionic plate with chorionic villi that project out. These are the largest volume of ECM among all the compartments of the placenta. The ECM of the stroma of the placental villi includes a collagen-based structure (predominantly types I, III & IV) which has both zonal and variable expression to support vascular capillary beds; adhesive glycoproteins such as laminin and fibronectin; and GAGs, specifically heparan sulphate. Upon decellularization, the remaining ECM maintains many of the pro-regenerative growth factors associated with cellular development, including VEGF, HGF, PDGF & TGF-β [8] and can be fabricated into both an injectable hydrogel and/or scaffold material to be used in applications such as skin substitutes, heart repair, and small diameter vascular grafts.

As a result of the various anatomical structures present within the placenta, it is possible to generate ECM scaffolds with a wide range of mechanical and biological properties to accommodate a multitude of applications in regenerative medicine. Beyond its structural ECM, the placenta serves as a rich reservoir of cellular and soluble therapeutic factors.

**Table 1:** Summary of Placental Compartments and ECM Properties.

Compartment	Vascularity	Key Structural ECM Components	Major Bioactive Factors	Primary Target Applications	References
Amniotic Membrane (AM)	Avascular (passive diffusion)	Laminin, Collagen types I, III, IV	HGF, EGF, bFGF, TGF-β isoforms	Cornea, cartilage, wound care, ocular surface reconstruction	[24,25]
Chorion	Highly vascularized	ECM-rich stroma	ANG, ANG-2, bFGF, VEGF-A, HGF, IGF-1, PDGF (AA/BB)	Vascular grafts, constructs with high metabolic demand	[19,20]
Umbilical Cord (Wharton’s Jelly)	Matrix itself is avascular, but encapsulates the umbilical vessels	Proteoglycans, hyaluronic acid, Collagen types I, III, V, VI	IGF-1, PDGF, TGF-β, bFGF, EGF, VEGF	Hydrogels for lubrication, cushioning, space filling, joint repair	[21,26]
Placental Disc	Highly vascularized	Collagen types I, III, IV, laminin, fibronectin, heparan sulphate	VEGF, HGF, PDGF, TGF-β	Skin substitutes, heart repair, small diameter vascular grafts	[27]

### 2.2. Placenta-Derived Cellular and Soluble Therapeutic Components

The placenta is one of the most prominent sources of MSCs, which are obtained post-partum without invasive procedures for the donor [28]. MSCs are found in many regions within the placenta, including the AM, chorionic plate/villi and WJ. The AM yields cells that originate from the fetus, while the chorionic plate/villi yield cells of both maternal and fetal origin [29]. Studies comparing these sources have identified significant differences among them: when cultured under serum-free conditions, MSCs derived from the chorionic plate (CP-MSCs) were capable of superior proliferation compared with MSCs derived from the AM (AM-MSCs) and umbilical cord (UC-MSCs) (validated via Colony-Forming Unit and Cell Counting Kit-8 assays at passage 5). Beyond proliferation, differential gene expression profiling reveals that these anatomical origins dictate distinct functional signatures that can be strategically aligned with specific therapeutic uses [30]. Specifically, CP-MSCs uniquely upregulate genes associated with cytokine production and inflammatory responses, making them highly suited for immunomodulatory therapies. In contrast, AM-MSCs are primed for biological adhesion, making them ideal candidates for epithelial reconstruction and scaffold integration, whereas UC-MSCs show distinct enrichment for cardiovascular system development and cell motility, positioning them as the optimal choice for vascular tissue engineering. Beyond these compartment-specific advantages and their broad differentiation potential across multiple lineages (including osteocytes, adipocytes, and chondrocytes), placental MSCs generally exhibit higher rates of proliferation, motility, and viability compared to adult sources like bone marrow [29]. They also actively suppress T-cell proliferation, thus reducing the likelihood of immune rejection in allogeneic transplantations [31,32].

A critical cell-free approach to utilizing placental therapeutics is the preparation of Human Placental Extracts (HPE). Unlike structural scaffolds or living cell populations, HPE is a highly concentrated, biologically active lysate produced by breaking down placental tissue. The resulting formulation contains low-molecular-weight peptides, amino acids, vitamins, minerals, and concentrated growth factors. Because it captures the potent, native secretome of the placenta in an easily administrable form, HPE exhibits substantial and unique pharmacological activities, driving targeted anti-inflammatory, analgesic, neuroprotective, and regenerative responses [33,34]. Clinically, this specific biochemical profile has proven highly effective across diverse systems: in liver dysfunction, Yamauchi et al. demonstrated that HPE suppresses oxidative stress and fibrosis in non-alcoholic steatohepatitis (NASH) models [35]. Its unique regenerative capacity also extends to musculoskeletal tissues, improving ligament healing [36], and provides neuroprotection by reducing neurological symptoms in multiple sclerosis models via the regulation of IL-23 and IL-27 levels [37].

The major biological advantage of these extracts over isolated or synthetic growth factors is that HPE acts as a comprehensive, synergistic molecular ‘cocktail’. Prepared through lytic or hydrolytic processes, these extracts yield several bioactive compounds [38]. For instance, Tonello et al. identified over 2000 distinct molecules in placental matrix fractions, including angiogenin and Bone Morphogenetic Protein 1 (BMP-1) [39]. Similarly, commercial hydrolysates like Laennec provide rich peptide and amino acid profiles alongside nucleic acids and a broad spectrum of native growth factors (VEGF, PDGF, bFGF, TGF-β, EGF) [36]. Another key component driving this regeneration is polydeoxyribonucleotide (PDRN), which supports nucleic acid synthesis [40].

Crucially, the processing method and final physical format of the matrix—such as the transition from a fluid lysate to a dehydrated powder—dictate how these native factors are delivered to the tissue. For instance, Koob et al. demonstrated that micronised dehydrated Human Amnion/Chorion Membrane incubated in saline for 24 h released 4–62% of its quantified growth factors (lowest for PDGF-BB, highest for EGF), reflecting a rapidly released unbound fraction alongside tissue-bound factors available for sustained release during matrix remodeling [41]. This dual-release profile was associated with 3- to 4-fold increases in human dermal fibroblast proliferation in vitro and enhanced mesenchymal progenitor cell recruitment in a mouse skin-flap model, consistent with paracrine signaling [41]. The regenerative potential of these complex, dehydrated placental cocktails can be harnessed into bio-inks [42] and controlled-release microparticles [39] to sustain vascularization in engineered constructs.

Following the use of bulk tissue extracts, another advanced cell-free approach involves Placental Extracellular Vesicles (PEVs). PEVs are lipid-bound nanoscale particles, typically isolated from placental tissue perfusates or the conditioned media of cultured placental cells using techniques such as ultracentrifugation or size-exclusion chromatography. While the aggressive processing required to create bulk lysates or decellularized matrices can sometimes result in the loss of delicate signaling peptides and native matrix properties, PEVs offer a protected, highly targeted alternative. Each vesicle encapsulates specific, concentrated combinations of bioactive proteins—often delivering discrete packages of 3 to 4 key signaling molecules that directly mimic natural paracrine transmission. By capturing this diverse, synergistic cargo naturally secreted by multiple placental populations (including MSCs and trophoblasts), PEVs provide a highly efficient therapeutic alternative to live MSC transplantation for their trophic properties. This approach enables to harness the placenta’s potent immunomodulatory and regenerative benefits without the safety risks and storage issues associated with live-cell therapies. Alternatively, these cell-free vesicles can be used as functionalizing agents, added directly into acellular scaffolds or bio-inks to enhance their regenerative capacity. There are three ways that PEVs can be isolated: (i) ultracentrifugation; (ii) polymer-based precipitation methods that do not require the use of centrifuges to obtain EVs; and (iii) size-based filtration that separates larger particles from smaller ones [43,44,45]. PEVs represent a highly heterogeneous population of membrane-bound particles that include larger microvesicles (100–1000 nm) and apoptotic bodies (>1000 nm); however, the therapeutically active fractions most commonly isolated and studied for regenerative applications fall predominantly within the smaller exosomal range. For instance, according to Zabel et al., the vast majority of isolated PEVs range in diameter from approximately 40 to 150 nm. Consistent with this exosome-dominant size profile, they also highly express traditional exosomal markers such as CD63 [46]. PEVs can deliver active cargo (such as proteins, cytokines, and miRNA), allowing for the transfer of bioactive cargo between various cell types. This enables protection of damaged cells and could aid their regeneration. As an example, Zhou et al. were able to stimulate endogenous neurogenesis and promote axonal outgrowth in rat spinal cord injury models through the administration of exosomes derived from rat placental MSCs [47]. Additionally, there is evidence that PEVs can induce angiogenesis regardless of the cell type that produces them. Komaki et al. have shown that PEVs significantly increase both endothelial tube formation and vascular gene expression in both in vitro and in vivo systems [48]. In addition to supporting vascular development and protection of the nervous system, PEVs exhibit many other immunomodulatory actions, making them suitable for the treatment of various inflammatory conditions. Perinatal-derived EVs affect both the innate and adaptive immune responses—inhibit Th1 and Th17 differentiation, promote the generation of regulatory T cells, decrease B-cell proliferation, and influence macrophage polarization to an anti-inflammatory M2 phenotype [49]. The immune-modulation-based mechanisms are responsible for the therapeutic efficacy observed in various models of disease including liver fibrosis, lung fibrosis, collagen-induced arthritis, experimental autoimmune encephalomyelitis, and cerebral ischemia [49]. Importantly, the immunomodulatory activity of perinatal cells is generally derived from the secretome (soluble factors and cargo within EVs) and not through cell-to-cell contact. Therefore, using perinatal EVs as a cell-free therapeutic agent could provide a method of mimicking the favorable effects of MSC transplantation on inflammation while avoiding the associated risks [49,50]. Thus, the delivery of PEVs via injectable and/or scaffold-integrated formats provides a promising cell-free approach for controlling vascularization and repairing tissues in engineered constructs.

Compared to whole-cell therapies, the advantages of using PEVs include reduced immunogenicity, lack of replication risk, and increased suitability for standard storage and delivery [51]. These features can be advantageous for clinical-grade biofabrication, where the creation of consistent batches and formulation that can be stored at room temperature is critical. One obstacle to widespread clinical use of PEV-based therapies is the lack of standardized protocols for the isolation, characterization, and potency assessment of PEVs. Another barrier is the lack of understanding of how variations in the cargo of EVs—which vary depending upon the health status of the donor, gestational age, and pathology—impact the subsequent therapeutic efficacy of the PEVs [51,52]. Addressing these gaps in dosing, storage stability, and manufacturing reproducibility will be essential for the clinical translation of PEV-based regenerative strategies.

## 3. Decellularization Methods

To harness the structural benefits of the placental ECM while minimizing the risk of immune rejection from native cells, the tissue must be decellularized. In general, a decellularization protocol requires a sequential approach (Table 2).

Physical Decellularization: The initial step of cell removal often relies on physical disruption, though the specific technique must be carefully tailored to the desired final format of the placental scaffold. For instance, continuous mechanical agitation (typically via orbital shakers or magnetic stirring) is widely used to facilitate the penetration of detergents such as sodium dodecyl sulfate (SDS) or Triton X-100 and osmotic solutions into intact placental tissues—such as the amniotic membrane—without destroying their delicate macro-architecture [53]. In contrast, homogenization—which involves high-shear force or bead-milling—is strictly reserved for creating pulverized or injectable placental matrices where the preservation of the native structural hierarchy is not required, such as in the formulation of flowable extracellular matrix hydrogels [54]. Additionally, freeze–thaw cycles are frequently employed to induce cell lysis via the formation of intracellular ice crystals. However, when used as a standalone method, freeze–thawing is generally insufficient for complete decellularization, often leaving up to 88% of residual DNA trapped within the matrix while risking the disruption of native ECM protein crosslinking [55]. Consequently, these physical methods are rarely used in isolation and must be synergistically coupled with downstream chemical or enzymatic treatments to achieve the benchmark DNA threshold for decellularization [55].

Chemical Decellularization: To remove the cellular material lysed by physical methods, primarily ionic and non-ionic detergents are employed as the standard agents. While both types are used to remove cellular components, they differ significantly in their efficacy for nuclear material removal and their impact on the ECM ultrastructure. Ionic detergents, such as SDS, are harsher and can denature ECM proteins, remove GAGs, and leave behind cytotoxic residues. Non-ionic detergents, such as Triton X-100, are milder; Kafili et al. demonstrated that protocols using 1% Triton X-100 retained approximately 65% collagen and 48% GAG content [56]. While non-ionic detergents are less effective at removing nuclear material, it is common to combine the two methods [57,58]. Additionally, acids such as peracetic acid (PAA) are often used to sterilize the scaffolds [55,59].

**Table 2:** Comparison of Decellularization Strategies for Placental Tissue.

Decellularization Method	Specific Agents	Primary Mechanism	Advantages	Disadvantages	References
Chemical (Ionic)	SDS	Solubilizes cell and nuclear membranes	Highly effective at removing cellular and nuclear material	Denatures ECM proteins, removes GAGs, leaves cytotoxic residues	[42]
Chemical (Non-ionic)	Triton X-100	Milder solubilization	Better preserves ECM ultrastructure and a higher fraction of collagen and GAGs compared with SDS.	Less effective at removing nuclear material when used alone	[56]
Physical	Freeze–thaw; Perfusion systems	Ice crystal-induced lysis; fluid shear stress	Efficient distribution via vasculature (perfusion)	Freeze–thaw alone is insufficient and damages ECM; perfusion depends on tissue structure	[60]
Enzymatic	Thermolysin; Streptokinase	Proteolytic and fibrinolytic degradation	Removes specific cells/clots while preserving basement membranes for cell attachment	Prolonged exposure to enzymes like trypsin breaks down collagen and GAGs	[20,61]

Perfusion-Based Systems: Dynamic perfusion systems can be used to maximize the efficiency of these physical and chemical methods. For example, Duan et al. combined these approaches by creating a perfusion-based system to dynamically distribute SDS and nucleases throughout the placenta via its native vasculature [42]. This approach enabled highly efficient removal of cells. A drawback, however, is that efficacy depends heavily on the perfusion parameters and the specific structure of the tissue being treated. Studies comparing perfusion methods to static immersion methods have shown conflicting results based on these variables [60].

Enzymatic Decellularization: Following physical and chemical disruption, specific residual cellular materials can be targeted for degradation using enzymes such as proteases, nucleases, and esterases. For example, nucleases, such as DNase and RNase, are typically added to enzymatic decellularization protocols after detergent-induced cell lysis to eliminate immunogenic DNA fragments. To disrupt cell-to-cell adhesion and cell-to-ECM interactions, proteolytic enzymes, such as trypsin and dispase, are similarly added. Trypsin is typically used in combination with Ethylenediaminetetraacetic acid (EDTA) to separate cells, but prolonged exposure causes the breakdown of many ECM components, including collagen and GAGs [55]. For processing the AM, Hopkinson et al. showed that thermolysin (0.25 mg/mL at 37 °C for 30 min) was effective in removing epithelial cells without damaging the underlying basement membrane, while dispase or EDTA treatments were shown to cause significant damage to ECM integrity. Moreover, the preserved matrix was capable of supporting subsequent cell growth, thus demonstrating its potential as a substrate for tissue engineering [61]. Shariatzadeh et al. used fibrinolytic enzymes such as streptokinase to decellularize the amniochorionic membrane by dissolving the blood clot within the chorionic microvessels. This resulted in the removal of the cellular layers on each side of the membrane, leaving the amniotic and chorionic basement membranes exposed. Through Scanning Electron Microscopy, MTT assay, and Immunohistochemistry characterization, it was determined that these exposed surfaces significantly improved endothelial cell attachment and proliferation [20].

Established benchmarks to verify decellularization effectiveness indicate that the residual amount of DNA should be under 50 ng/mg of dried material. This value represents effective cell removal from the scaffold and demonstrates low immunogenic potential for the resulting scaffold [62]. Biomaterials derived from placentas have an inherent advantage in this respect: native placenta tissues express significantly lower levels of classical MHC antigens than other tissues [8]. Because decellularization is never fully complete, and residual membrane fragments inevitably remain trapped within the matrix, the intrinsically low MHC expression of placental tissue ensures that processed ECM exposes a markedly lower baseline immunogenicity compared to other tissue sources, even prior to optimizing processing conditions. The specific choice of decellularization protocol, alongside the duration the processed tissue is stored prior to use, is critical in determining whether the final biomaterial retains its native biological efficacy. Although aggressive decellularization protocols (e.g., those utilizing SDS) can achieve complete cellular removal, they risk the loss of native growth factors. Therefore, protocols must balance effective cell removal with the preservation of the ECM’s structural and bioactive properties—ultimately maximizing safety and therapeutic efficacy [60,63]. Moreover, preservation of mechanical, biophysical and angiogenic characteristics of decellularized tissues during storage is essential. For example, Asgari et al. demonstrated that the storage of the decellularized placental sponge (DPS) patch for 6 months in a −20 °C freezer can reduce the biological activity of the DPS patch to a significant degree [63]. The VEGF levels in the 1-, 3- and 6-month stored DPS remained stable (measured via ELISA), but the angiogenic potential of these patches decreased as indicated by fewer vascular junctions in CAM (chick chorioallantoic membrane) assays, and by fewer CD31+ cells in subcutaneously implanted mice with DPS at 6 months. Furthermore, the broad-spectrum antibacterial efficacy of the DPS against common pathogens (such as Staphylococcus aureus and Escherichia coli) progressively deteriorated, becoming completely absent after 3 months of storage. Coupled with a marked increase in CD4+ and CD68+ infiltrating immune cells at 6 months—indicating a loss of the scaffold’s immune-evasive properties and the onset of an active pro-inflammatory foreign body response—these findings demonstrate that a 3-month storage limit is optimal for maintaining the patch’s biocompatibility, angiogenic, and antibacterial activities [63].

## 4. Applications in Regenerative Medicine

Building upon their diverse structural and biochemical profiles, placenta-derived biomaterials are transitioning from simple biological dressings to sophisticated regenerative matrices. Before addressing their broader impact, we first examine well-established biomaterials that are already in clinical practice and their emerging roles in advanced tissue engineering and 3D biofabrication (Table 3).

These therapeutic benefits, however, should be interpreted with caution. Studies in this field vary widely in methodology. Outcomes expressed as percentage improvements and fold-changes drawn from disparate assays cannot be directly compared. Many of the promising results described below originate from small, single-animal in vivo experiments or early-stage ex vivo systems. Consequently, meaningful evaluation of differences between biomaterials is constrained by the limited statistical power and model variability, and these preliminary observations must be interpreted in light of the inherent limitations of the various experimental systems used to generate them.

### 4.1. Established Clinical Applications

**Figure 2:**
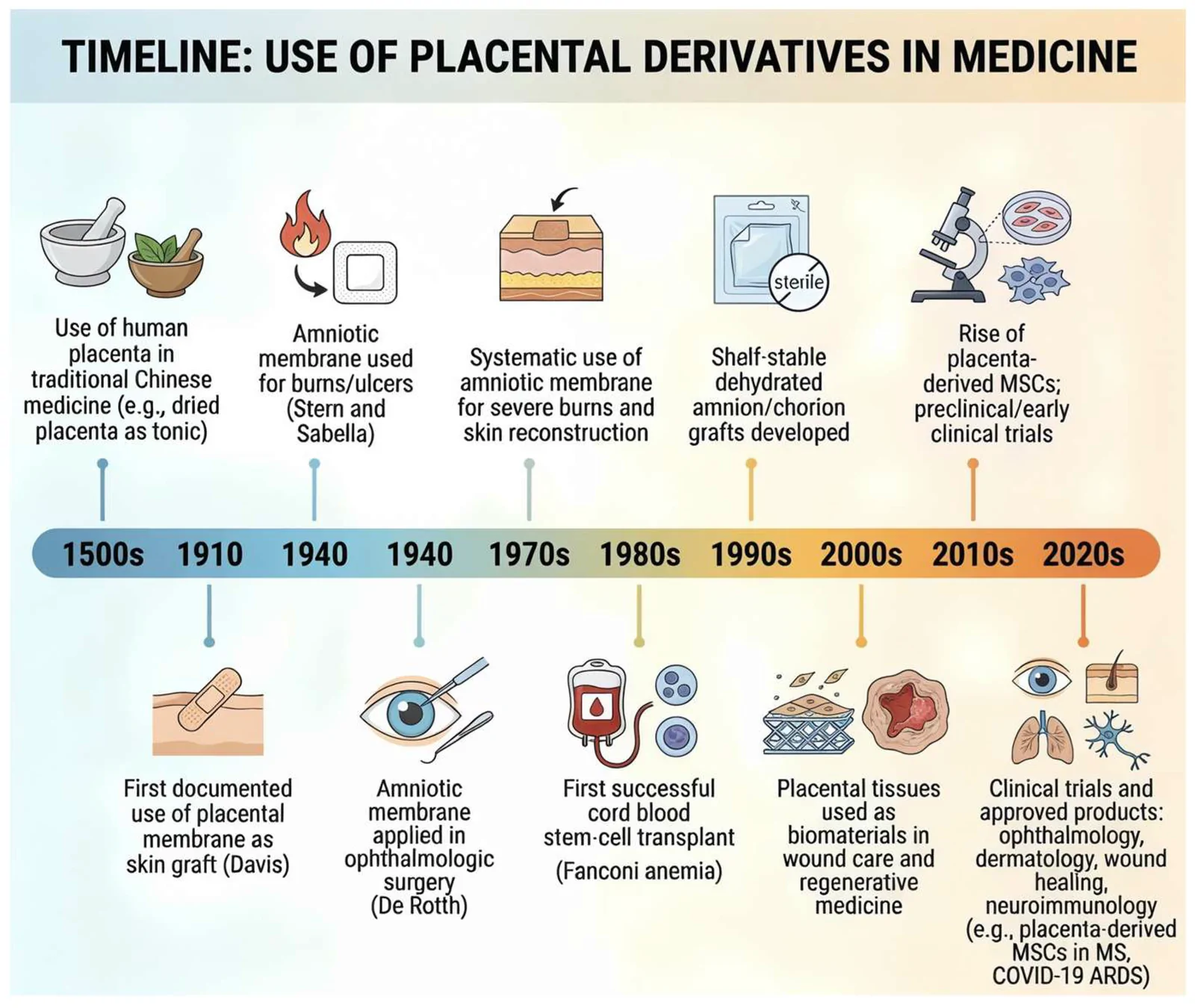
Horizontal timeline of selected clinical and translational milestones in the use of placental derivatives in medicine. From traditional and early surgical applications to modern regenerative and cell-based therapies, placental membranes, amniotic fluid, umbilical cord, and placental-derived stem cells have been progressively repurposed as biomaterials and bioactive products. MSC = mesenchymal stromal/stem cell; ARDS = acute respiratory distress syndrome [8,33,66]. Image generated by the authors.

From the simple wound patch first used in the early 1900s, commercial products have been developed for clinical use of placental tissues as shown in Figure 2. The modern clinical foundation for placenta-derived materials was built upon the success of the AM in ophthalmology. Its anti-inflammatory, anti-fibrotic, and low-immunogenicity properties proved highly effective for ocular surface reconstruction [17]. Building on this ophthalmic success, these properties have been widely translated into commercialized sheet-form scaffolds and particulate matrices for chronic wound care. For instance, a prospective randomized controlled trial by Zelen et al. compared dehydrated human amniotic/chorionic membrane (dHACM) to standard of care for diabetic foot ulcers, demonstrating a statistically significant increase in healing rates at both 4 weeks (77%) and 6 weeks (92%), compared to 0% and 8%, respectively [64]. Similarly, Tettelbach et al. performed a multicenter randomized controlled trial showing that dehydrated human umbilical cord (EpiCord) achieved significantly higher wound closure rates at 12 weeks than a collagen–alginate control (77% vs. 46%) [65].

### 4.2. Emerging Tissue Engineering Applications

Beyond clinical wound care, there are many specialized applications for non-printed acellular placental and perinatal matrices. For solid tissue engineering, the focus has shifted toward harnessing the unique architecture of native placental structures. For example, while historically chemically stabilized human umbilical vein grafts were used in vascular reconstruction, today’s focus in regenerative medicine has shifted toward decellularized umbilical vessels as highly biocompatible scaffolds for small-diameter, tissue-engineered vascular grafts [67,68].

### 4.3. Injectable and Flowable Matrices

In addition to solid scaffolds, there is growing interest in developing injectable approaches for minimally invasive delivery. For example, Francis et al. (2017) treated ischemic rat hearts with injectable hydrogels derived specifically from the decellularized placental disc (hpECM) [54]. Cardiac performance was assessed using echocardiography, and the extent of scar formation was determined using histologic sections and Masson’s trichrome staining. The authors found that hpECM injection resulted in significantly smaller infarct areas and better preservation of left ventricular wall thickness than the saline-treated group. Additionally, the hydrogel increased the metabolic activity of both stem cells and cardiomyocytes in culture, promoting the spontaneous contraction of the latter [54].

Alongside these hydrogels, flowable human AM-derived products are being investigated as adjuncts to enhance tendon repair in orthopedics. To assess the effects of processing techniques on matrix efficacy in promoting tenocyte growth, Mao et al. (2022) investigated the in vitro behavior of human tenocytes seeded onto three commercially available placental connective tissue matrices: a non-decellularized, minimally manipulated particulate matrix (AmnioFill^®^); a non-decellularized liquid matrix (BioRenew™); and a decellularized, flowable particulate matrix (Interfyl^®^) [69]. Direct comparison of these matrices allowed the authors to identify differences attributable to decellularization. Non-decellularized matrices provided limited support for tenocyte proliferation (BioRenew™ supported none at all). In contrast, Interfyl^®^ showed excellent cytocompatibility, providing an environment that supported tenocyte proliferation (doubling time ~3 days vs. 15 days for AmnioFill^®^). Additionally, tenocytes grown on Interfyl^®^ showed a marked increase in migration (*p* = 0.005 vs. control) and maintained their tenogenic phenotype based on mRNA expression of SCX, TNC, COL1A1, and COL3A1 as well as increased type I collagen protein. Lastly, Interfyl^®^ also appeared to modulate inflammation through decreased gene expression of pro-inflammatory cytokines (TNF-α, CXCL8/IL-8) and a matrix-degrading enzyme (MMP-1) under TNF-α stimulation when compared to AmnioFill^®^. Therefore, Interfyl^®^ likely limits dedifferentiation and fibrotic scar formation while creating a favorable environment for tendon regeneration through the increased expression of TGF-β3 [69].

**Table 3:** Recent Applications of Placental Biomaterials in Regenerative Medicine.

Biomaterial Format	Target Application	Key Biological Outcomes vs. Control	References
Dehydrated human amniotic/chorionic membrane (dHACM)	Diabetic foot ulcers (Clinical Trial)	77% complete healing at 4 weeks, 92% at 6 weeks (vs. 0% and 8% for standard of care)	[64]
Dehydrated human umbilical cord (EpiCord)	Wound closure (Clinical Trial)	70% wound closure at 12 weeks (vs. 48% for alginate control; ITT analysis)	[65]
Injectable hpECM hydrogel	Ischemic rat hearts (In vivo)	64% reduction in average infarct volume vs. saline; accelerated in vitro contraction (8 h vs. 2–4 days).	[54]
Decellularized flowable particulate matrix (I-CTM)	Tendon repair (In vitro)	5-fold increase in proliferation rate (doubling time ~3 days vs. 15 days) and significantly increased migration.	[69]

## 5. Placenta-Derived Bio-Inks for 3D Bioprinting

Placental ECM can be incorporated into bio-ink formulations to engineer large-scale, viable tissue structures. This bioactive additive helps achieve the critical balance required of an ideal bio-ink: optimal rheology for printability combined with the mechanical stability necessary to support cellular viability, attachment, proliferation, and lineage-specific differentiation. However, converting native placental tissue into a suitable bio-ink requires optimizing multiple chemical and physical properties.

### 5.1. Overcoming Mechanical Barriers in Placental Bio-Ink Engineering

**Figure 3:**
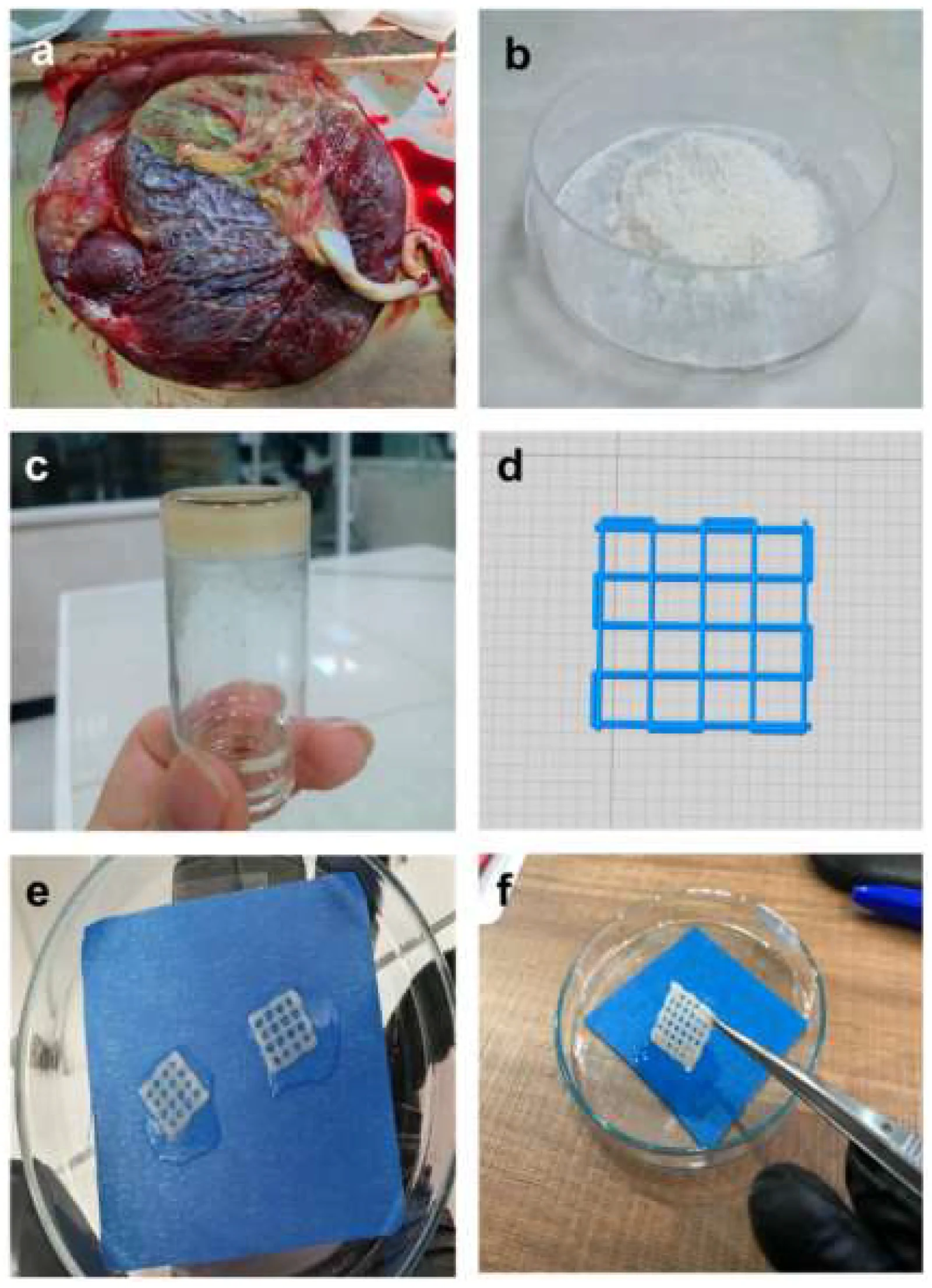
Workflow for the preparation and 3D bioprinting of placental extracellular matrix (ECM). The sequence illustrates the processing of raw human placenta (**a**) into lyophilized ECM powder (**b**) and its formulation into a viscous, printable bio-ink (**c**). Using a predefined digital grid model (**d**), the optimized bio-ink is extruded to yield structurally stable, 3D-printed hydrogel scaffolds (**e**,**f**). Reproduced with permission from John Wiley and Sons, License No. 6290730167014 [73].

The transformation of a placental ECM from a raw biological framework to a printable hydrogel involves a systematic progression through solubilization, rheological modification, and reinforcement of structure (Figure 3). Solubilization is generally accomplished using enzymatic degradation to transform the rigid matrix into a liquid precursor. Zhang et al. reported one major advantage of utilizing pepsin-digested decellularized ECM (dECM): the ability of the bioink to undergo entropy-driven self-assembly at body temperature and pH, allowing the liquid bioink to be transformed to a cross-linked stable gel simply by incubating without the need for potentially cytotoxic chemical cross-linkers [70]. Although enzymatic digestion remains the most common method of solubilizing placental ECM, Bashiri et al. showed that chaotropic agents (such as urea) are also viable alternatives. Urea does not break collagen bonds as some enzymes do, and therefore preserves the structural components of the placental tissue, which can be used to regenerate tissue [71]. After solubilization, the liquid precursors exhibit thermally responsive gelation. Duan et al. determined that human placental bio-inks stored at 4 °C gel within 20 min when heated to 37 °C, and that these gelation kinetics are important for cell encapsulation [42]. Pure dECM, however, exhibits poor shear-thinning properties, making it unable to be printed via extrusion methods. Bashiri et al. found a significant concentration threshold beyond which dECM becomes non-printable and fails to maintain filament integrity, particularly for dECM concentrations above 5% *w*/*v* [71]. To address this issue, Kafili et al. demonstrated that modifying the concentration of digested AM (from 1% to 3%) resulted in a higher storage modulus and viscosity of the dECM solutions, enabling high-resolution printing that would collapse at lower concentrations [56]. As a result, composite approaches have been developed to solve these issues. Specifically, Bashiri et al. utilized a silk fibroin/alginate backbone to provide structural support for placental ECM [71], while Pazhouhnia et al. (2024), employing extrusion-based 3D bioprinting, engineered a bilayered scaffold by incorporating 0.1 mg/mL of amniotic membrane extract (AME) into a 15% gelatin methacrylate (GelMA)/10% gelatin hydrogel [72]. The gelatin component provided mechanical stability to the bilayered scaffold, achieving an average elongation at break of 56% and enhancing the Young’s modulus (*p* < 0.05) compared to pure GelMA. Additionally, the incorporation of placenta-derived AME significantly improved the viability of HUVECs according to live/dead staining, while RT-qPCR analysis demonstrated that it increased the transcriptional expression of VEGF, thereby enhancing angiogenic processes [72]. Finally, the composite structure supported the development of separate, phenotypically distinct layers of skin: the gelatin component specifically upregulated Keratin 10 (KRT10) in keratinocytes—a key marker of epidermal differentiation—and Vimentin (VIM) in fibroblasts, indicating robust dermal matrix production and cellular maturation [72].

### 5.2. Optimization of Printing Variables

The successful three-dimensional (3D) bioprinting of a placenta bio-ink depends upon the simultaneous optimization of several interdependent variables. It is important to note that while many of these factors are universal bioprinting principles applicable to any hydrogel, they must be specifically calibrated to the unique rheological properties of the pECM. As a general rule in extrusion-based bioprinting, the nozzle size (0.2–1.0 mm) significantly affects the resolution of the printed layers and the shear force exerted on the cells. Smaller nozzle sizes allow for better resolution; however, this can be at the cost of cell viability due to higher shear forces [74,75]. Similarly, universal kinetic variables such as print speed (4–10 mm/sec) and extrusion pressure (100–300 kPa) must be carefully balanced. Print speeds that are too high cause irregularities in filament deposition, whereas print speeds that are too low result in over-extrusion and loss of structural detail [76,77].

The concentration of the ECM and its thermal properties are the defining, tissue-specific factors. When applying these general principles specifically to placental bio-inks, higher concentrations of placental ECM (typically >10 mg/mL) provide greater structural integrity and print fidelity; however, this also results in higher viscosities that may exceed the optimal range for extrusion [78,79]. Furthermore, temperature control is highly specific to the biological source: while standard bio-inks may have different thermal windows, most placental bio-inks require specific temperatures of 15–25 °C during printing to remain fluid and prevent premature gelation [80]. Standard cross-linking strategies (temperature-mediated, chemical via EDC/NHS or genipin, and photo-polymerization) are utilized, but they must be carefully tailored to the density of the placental ECM to ensure rapid shape retention while maintaining sufficient pore size for cellular migration and nutrient diffusion [78,81].

Ultimately, these variables are highly interdependent; for example, an increase in the concentration of the placental bio-ink will require a corresponding increase in the extrusion pressure and adjustments to the nozzle size. Due to the complexity of this process, computational models and designs of experiments are being developed to optimize the formulation and printing of these advanced tissues [82,83].

### 5.3. Enhancing Biological Functionality and Biomimicry

The work of developing bio-ink does not just involve the development of mechanically printable biomaterials but also includes enhancing structural stability and biological function through the use of chemical modification (such as to enable controlled crosslinking), controlled release mechanisms, and in vitro microphysiologic models of tissue architecture. One common way to improve the stability of natural matrices is photo-crosslinking via chemical modifications, such as adding methacryl anhydrides to dHAM (dHAMMA), a cross-linkable version of the dHAM matrix. A bicomponent network that included dHAMMA cross-linked with GelMA exhibited a tensile strength that was 18-fold stronger than the non-modified dHAM. Furthermore, this material improved wound healing in mice compared to untreated wounds [84]. Another method to improve the functionality of synthetic scaffolding materials is to add bioactive signals to the scaffold through the addition of decellularized placenta powder (PP) to synthetic polymers such as poly(ethylene glycol)-based hydrogels through a UV-curable thiol-ene reaction. The addition of 1–8 wt% of PP to the hydrogel added collagen-rich bioactive signals that allowed the cells to adhere to the surface of the hydrogel and maintain viability at levels >91% for both human skin fibroblasts and murine macrophage cells. Additionally, the inclusion of PP into the hydrogel created a filler effect that reduced the swelling ratio and significantly increased the stiffness of the hydrogel, resulting in a range of tunable storage moduli that ranged from 1080 ± 290 Pa to 51,400 ± 200 Pa [85]. Alongside the advancements in structure and properties of biomaterials, Tonello et al. have developed methods to stabilize labile growth factors such as VEGF and angiogenin by encapsulating the soluble form of human placental matrix into PLGA microparticles. The microparticles demonstrated high encapsulation efficiency (approximately 75%) and delivered VEGF and angiogenin over 30 days, where a total of 60–70% of the encapsulated growth factors were delivered during this time frame. Functional studies showed that the released matrix stimulated a two-fold increase in endothelial cell proliferation and enhanced tube formation compared to the free matrix control [39].

The findings discussed above validate the potential for developing tissue constructs capable of forming complex tissues. In the context of vascularization, Heidari et al. employed a coaxial bioprinting approach using a bio-ink composed of an optimized mixture of 1% *w*/*v* alginate and 0.6% *w*/*v* ddHAM. The dHAM incorporated essential RGD motifs to enable HUVEC self-assembly into a mechanically stable construct (Young’s modulus = 32.35 ± 5 kPa) that increased cell viability by 492 ± 18.8% and enhanced VEGFR-2 expression by 14 days [86]. Consistent with these findings, ex vivo models further demonstrate the potent angiogenic capacity of placental-derived bio-inks. Chorioallantoic membrane (CAM) assays reveal that 3D-printed alginate/gelatin constructs supplemented with 5% placental ECM (5%ECM-Alg/Gel) show a significant increase in new blood vessel formation after 7 days compared to ECM-free controls (Alg/Gel) [80].

Wei et al. investigated molecular drivers of osteogenesis by employing LC-MS proteomic analysis to identify 1076 human placental-derived proteins (hPDPs) that include key stimulators such as GDF15 and SPARC. Their 10% hPDP gel significantly outperformed standard osteogenic media, producing a 19.3-fold increase in BMP2 and a 3.9-fold increase in COL1A1 mRNA expression in human mesenchymal stem cells. The authors attribute this enhanced potency to the gel’s ability to simultaneously activate the BMP and TGF-β signaling pathways, which translated into superior in vitro mineralization and distinct osteoid accretion in ectopic mouse models [87]. Similarly highlighting molecular potency, Khosrowpour et al. developed a decellularized placental sponge (DPS) capable of downregulating inflammatory markers (NOS2 and CD86) in vitro. When tested in critical-sized rat calvarial defects, the DPS produced significant osteogenesis—yielding 15.2% new bone formation compared to 10.83% in controls after 8 weeks—with immunostaining confirming a high density of tissue-remodeling CD206+ M2 macrophages in the defect area [88].

Despite these in vivo successes, a major bottleneck in translational tissue engineering remains the need for robust experimental models to evaluate biomaterials and complex cellular interactions prior to animal testing. To overcome this limitation, bioprinting is increasingly utilized to construct sophisticated in vitro microenvironments. For example, Sun et al. utilized digital light processing (DLP) to engineer a ‘placenta-on-a-chip’ that accurately mimics the maternal–fetal interface. Their tri-coculture design featured a 3D-printed hydrogel scaffold (18 kPa stiffness) encapsulating primary stromal fibroblasts, overlaid with human trophoblast stem cells, and perfused with endothelial cells at 3.5 µL/min. This construct successfully replicated physiological transport by limiting the diffusion of large molecules approximately 1.5-fold more than acellular controls, while producing key pregnancy hormones like estradiol and hCGβ [89]. Building on this concept for disease modeling, Ghorbanpour et al. developed a 3D microfluidic model to study preeclampsia. By co-culturing endothelial cells and trophoblasts, their platform accurately recapitulated human placentation dynamics that standard monocultures missed; notably, inducing a biphasic TNF-α response and enabling a six-fold increase in trophoblast migration under simulated inflammation [90]. Ultimately, the necessity of these complex bioengineered microenvironments was highlighted by Sayres et al., who attempted to rescue the inherent migratory deficiencies of primary endothelial cells derived from severe fetal growth restriction (FGR) placentas using standard 2D in vitro models coated with individual ECM substrates (fibronectin, laminin, collagens). Although some proliferative defects were mitigated, the continued presence of migratory dysfunction demonstrated the ineffectiveness of these simple, flat protein coatings. Accurately modeling or rescuing these permanently altered cellular phenotypes requires fully replicating the complex mechanical and biochemical environment of the native placental stroma, further emphasizing the need for multi-component bio-inks [91].

**Figure 4:**
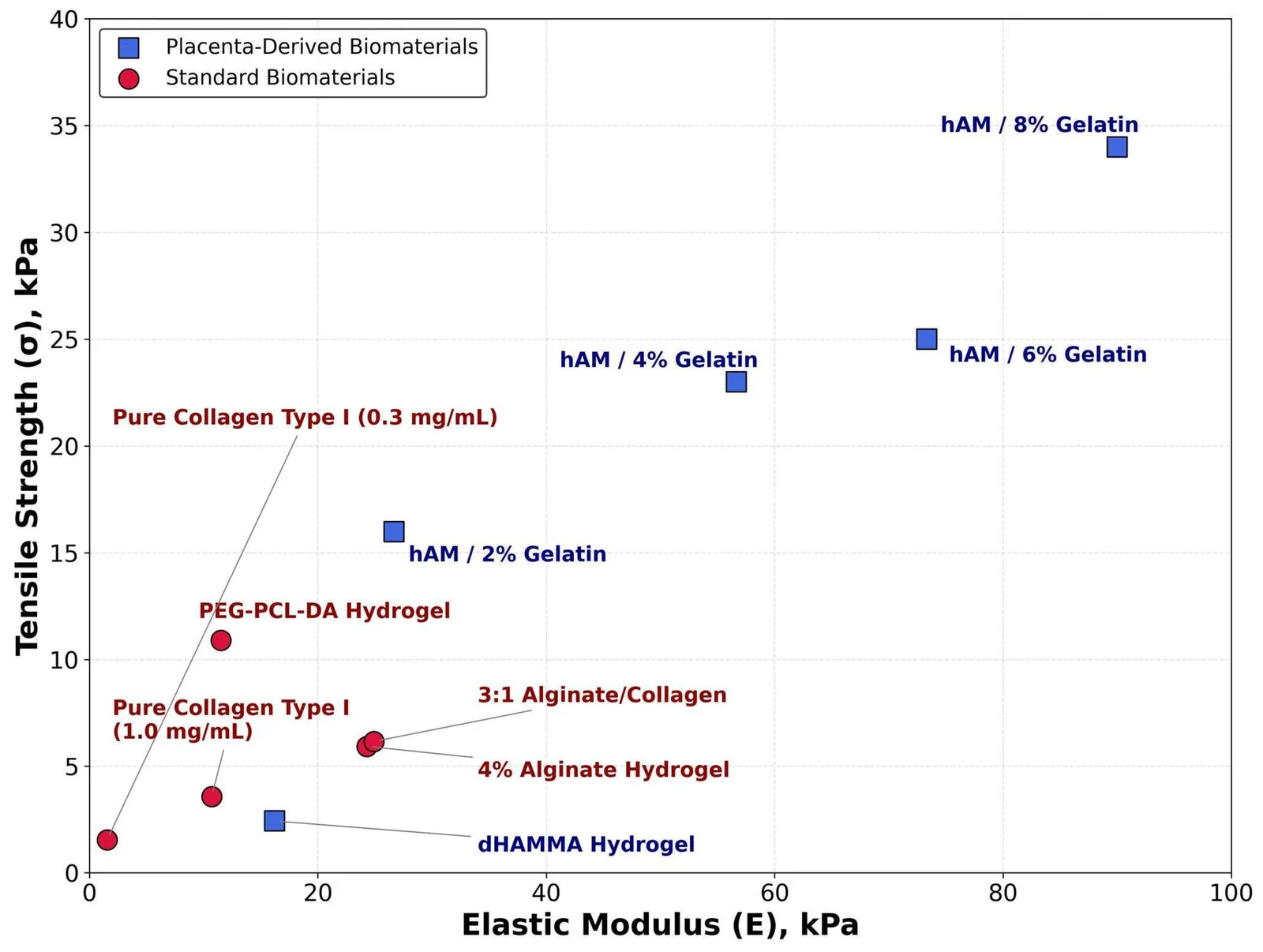
Mechanical phase-space plot of elastic modulus (E) versus ultimate tensile strength (σ) for placenta-derived biomaterials compared to standard biomaterials. Placenta-derived biomaterials (blue squares) comprise the dHAMMA hydrogel [84] and human amniotic membrane (hAM) formulations blended with varying gelatin concentrations (2–8% *w*/*v*) [99]. Standard biomaterial benchmarks (red circles) represent experimental reference values extracted from literature for soft-tissue hydrogels, including Pure Collagen Type I (0.3 and 1.0 mg/mL) [100], PEG-PCL-DA, 4% [101], Alginate, and 3:1 Alginate/Collagen [102]. The plot demonstrates the broad mechanical tunability of placental-derived matrices, spanning from soft, highly compliant formulations to stiffer hydrogel constructs within the soft-tissue biomechanical range.

Although the mechanical properties of placental ECM are well-documented [92], their practical utility is best appreciated when directly benchmarked against established soft-tissue biomaterials. An effective way to visualize this is by plotting the materials in a two-dimensional phase space of elastic modulus versus tensile strength [93]. When placenta-derived biomaterials, as detailed in Table 4, are mapped into this space alongside standard natural and synthetic hydrogels, placental formulations exhibit broad and customizable mechanical profile. Rather than being confined to a narrow structural range, they can be precisely tuned to match or surpass the mechanical integrity of conventional scaffolding materials (Figure 4).

## 6. Clinical Translation and Ethical Considerations

The primary barrier to translating placental biomaterials from benchtop to bedside is the regulatory classification of new products for which no safety and efficacy data exist. This implies the standardization of the entire process, from procurement of the placenta to the manufacture of the final product.

Currently, standard operating procedures lack consistency across procurement, decellularization, bio-ink formulation, and printing techniques [91,103]. This inconsistency creates significant difficulties in establishing reliable comparisons among studies.

Researchers screen placental samples for infectious diseases (e.g., HIV, hepatitis, West Nile virus, syphilis) prior to tissue processing and obtaining informed consent from the donor. Placentas obtained from cesarean section births are preferred over placentas obtained from vaginal deliveries due to lower levels of contaminants [104]. Some placental-derived products have received FDA approval for clinical use. For example, decellularized human AM has received FDA approval for wound dressing products (AmnioGraft, EpiFix); human placental tissue hydrogels are currently in clinical trials for wound healing and tissue repair applications [92,105].

Typically, the translation pathway for placental biomaterials requires demonstration of sterility, biocompatibility testing according to ISO 10993 [106], and compliance with GMP regulations for manufacturing [107,108].

Under Section 361 of the Public Health Services Act, the FDA exempts placental products from premarket approval, provided they are “minimally manipulated” and intended for homologous use [105,109]. However, rigorous processing methods essential for bio-ink development—such as decellularization or enzymatic digestion—fundamentally alter the native tissue, reclassifying these biomaterials as “more than minimally manipulated”. Similarly, applying placental scaffolds to cartilage or bone reconstruction constitutes a “non-homologous use” (Table 5). Consequently, products like powdered or flowable amniotic matrices often fall outside Section 361. In the U.S., placental biomaterials may be regulated as 361 HCT/Ps if they meet minimal manipulation, homologous use, and related criteria; otherwise, they may be regulated as biologics (triggering a lengthy Biologics License Application [BLA] process), devices, or combination products depending on product characteristics [105,109]. This complex landscape is mirrored in Europe. In the EU, viable cell-containing constructs are more likely to fall under Advanced Therapy Medicinal Product (ATMP) rules, requiring centralized approval, whereas non-viable decellularized products may fall under the strict Medical Device Regulation (MDR) depending on their intended use and classification. Ultimately, the profound expense and regulatory uncertainty across both jurisdictions heavily stifles innovation. Despite the distinct clinical advantages of injectable or cell-laden allografts for complex wounds, companies are financially pressured to limit their development to simple, acellular sheet scaffolds [92,105,110].

### 6.1. Safety, Biocompatibility, and Scale-Up

Scaling these therapies to commercial levels remains significantly hindered by the inherent biological variability of individual placentas, driven by differences in genetics, maternal health, and gestational age. While pooling tissue from multiple donors is a logical strategy to standardize product composition, implementing this at an industrial scale introduces severe logistical hurdles. Unlike standard blood banking, harvesting solid perinatal tissues requires a highly coordinated, time-sensitive cold-chain infrastructure to transport the tissue from the delivery room to the processing facility before the extracellular matrix begins to degrade [92]. Furthermore, its utilization in clinical applications is governed by strict safety frameworks. As is mandatory for any human-derived allograft, standard tissue banking protocols dictate that donors must be rigorously screened for infectious diseases (such as HIV, Hepatitis B and C, and Syphilis) prior to donation. These universal safety requirements, while well-established and routinely managed by modern tissue banks, must be seamlessly integrated into any mass manufacturing pipeline.

### 6.2. Ethical and Socioeconomic Considerations

Although placental tissue is generally classified as medical waste in many jurisdictions, its commercialization and use in research introduce unique bioethical and economic tensions. Under current international regulatory frameworks, the financial compensation of donors for human tissue is strictly prohibited. For products already in clinical use, the raw material is obtained exclusively through altruistic, unpaid donation following rigorous informed consent—often acquired prior to scheduled Cesarean sections. Because these discarded tissues are freely donated by the patient but subsequently processed, patented, and sold for profit by commercial entities, a complex economic paradox arises. Establishing transparent, standardized collection networks that respect this altruistic origin is critical.

Beyond these economic factors, researchers and regulatory bodies must actively navigate complex issues peculiar to perinatal tissue, such as its dual maternal–fetal genetic makeup and the sensitive timing required for obtaining consent. For instance, the development of complex placental models through tissue engineering significantly amplifies these informed consent challenges. Because the cells used to generate advanced constructs like trophoblast organoids can be frozen and stored for years, the ethical and genetic implications of their use extend long after the physical tissue has been donated. Chief among these concerns is the fact that placenta-derived materials inherently contain the genetic material of the fetus. This fetal genetic identity means that maternal consent alone is technically insufficient because the offspring is also a genetic stakeholder in the material [111].

The inability of the offspring to provide consent at the time of collection creates a ‘temporal consent gap.’ Current recommendations for managing this delay include re-contacting the offspring once they reach decision-making capacity, which varies from 12 to 18 years depending on the jurisdiction. However, in order to obtain informed consent for longitudinal studies or studies that involve biobanking of placental-derived materials for future, as-yet-undetermined uses, both parents (not only the mother) must be involved in the consent process.

A further issue that arises when considering the consent for placenta-derived models involves whether genetic information collected from placental tissue will have implications not only for the offspring but also for their biological relatives—similar to the “Family Member Problem” identified in genomic biobanks. While current international and national Institutional Review Board (IRB) guidelines provide some direction for managing consent for placenta-derived models, there is no uniformity across jurisdictions, and therefore, there is a critical need for regulatory agencies to develop additional guidelines in conjunction with advancements in the science.

## 7. Conclusions

The placenta has enormous potential for regenerative medicine because it is an abundant and ethically acceptable source of cells, biologics and ECM. Each placenta contains structurally and functionally similar biological components (decellularized ECM, MSCs and EVs) to those found in natural biological systems. These identical biological components of each placenta have been recognized as ideal biological resources for the development of new biomaterials for regenerative medicine.

Each placental biomaterial has unique and complementary therapeutic properties that, when used in conjunction with advances in 3D bioprinting and biofabrication platforms, provide viable paths toward constructing human tissues that match the compositional and architectural complexity of their native counterparts. Crucially, placental biomaterials address a major barrier in regenerative medicine by providing a fully human, and therefore xeno-free, alternative to standard animal- and murine sarcoma-derived basement membrane extracts (e.g., Matrigel). By eliminating the risks of xenogeneic immunogenicity and zoonotic transmission associated with these traditional in vitro matrices, placental materials establish the native human microenvironment that is essential for the successful clinical translation of regenerative products.

Realization of the full potential of placental biomaterials will require simultaneous advancements in three primary areas: creation of vascularized, multi-cellular constructs; scale-up of GMP-compliant production; and generation of clinical evidence through well-designed studies. As the complexity of placental-derived models increases and the genetic implications associated with the use of such models become increasingly relevant, the ethical framework supporting their use must also evolve in a manner consistent with the scientific advancements. Together, these advancements establish placenta-derived biomaterials as key components of the emerging xeno-free biofabrication paradigm.

## 8. Future Directions

Establishing consistent manufacturing processes is a high priority for the field. Currently, there is no universally accepted process for producing placental biomaterials that satisfies the needs of regulatory agencies while ensuring reproducible function of the material produced. Consistent standards for decellularizing, sterilizing, testing the quality of, and storing these types of biomaterials are necessary for any widespread clinical application. Beyond manufacturing, a major scientific challenge lies in how these biomaterials are subsequently evaluated in vitro. To more accurately model tissue interactions, there is a need to transition away from cancer-derived cell lines (such as immortalized choriocarcinomas), shifting toward primary or stem-cell-derived trophoblasts that better represent the native maternal–fetal barrier. However, simulating in vivo reality remains inherently complex. Any cell population capable of robust expansion on artificial culture substrates is prone to phenotypic adaptation. Consequently, the field’s focus must extend beyond simply changing the cell source; it requires rigorous experimental design. In fact, the limitations of the in vitro models used should be emphasized and matched with precise physiological controls. This also implies that the conclusions drawn from these isolated systems should be contextualized. Standardized methods for isolating, characterizing, and testing the potency of placental-derived exosomes are also urgently required to enable dose–response studies and comparisons among laboratories. To address these critical bottlenecks, international consortia are actively working to establish unified guidelines, notably the European COST Action SPRINT (International Network for Translating Research on Perinatal Derivatives), which aims to standardize the nomenclature, processing, and therapeutic application of these materials.

Building upon standardized materials, the field must evolve from simple, single-component scaffolds toward highly customized, multi-cellular constructs tailored specifically for their intended clinical application. Rather than simply attempting to recreate native placental tissue, the goal is to leverage the placenta’s unique properties to mimic the spatial and functional complexity of the final target tissue. Achieving this requires the integration of established bioprinting technologies with validated placental biomaterials. Furthermore, these advanced, multi-component bio-inks can be deployed to develop sophisticated in vitro testing systems, such as “placenta-on-a-chip” models, which are highly capable of supporting both drug testing and pathophysiology-related research. However, a major obstacle to advancing volumetric grafts—whether for in vivo implantation or complex in vitro modeling—is the engineering problem of developing functional, perfusable vascular networks. This issue has not yet been resolved and currently limits the clinical utility of all thick, three-dimensional biomaterial constructs.

Ultimately, bridging the translational gap between basic research and clinical practice requires the generation of robust clinical evidence. Currently, there are few, if any, comparative, randomized clinical trials that have evaluated the effectiveness of placental biomaterial-based constructs compared to standard-of-care treatments. Generation of this type of evidence will be essential for obtaining regulatory approvals and for gaining widespread acceptance of these biomaterials in the clinical setting. Realizing this goal will require sustained interdisciplinary collaboration at a scale that individual research groups cannot achieve alone.

## Figures and Tables

**Table 4:** Mechanical Properties of Placenta-Derived Bio-inks vs. Native Target Tissues.

Biomaterial Formulation	Reported Mechanical Property	Target Application	Native Target Tissue Property	References
Amniotic Membrane Hydrogel (dHAMMA)	Tensile strength (maximum load) of 2.43 ± 0.09 kPa	Skin defect healing	7.2–10.7 kPa (Complex modulus via dynamic indentation)	[84,94]
GelMA/Gelatin/AME (0.1 mg/mL)	56% elongation at break	Skin tissue engineering	27–70% (Ultimate strain/elongation at break, highly dependent on orientation and age)	[72,95]
PEG Hydrogel + Placenta Powder	Tunable storage modulus of 1.08 ± 0.29 kPa to 51.4 ± 0.2 kPa	Soft tissue filling	3.2–14.9 kPa (Includes Young’s modulus for human breast fat and shear modulus for subcutaneous adipose tissue; dependent on temperature and testing conditions)	[85,96]
3D-printed Placenta-on-a-chip	18 kPa stiffness	Maternal–fetal barrier	1.25 kPa (Decidua basalis); 0.17 kPa (Decidua parietalis); 0.23 kPa (Placenta)	[97,98]

**Table 5:** Regulatory Pathways and Classifications for Placental Biomaterials.

Jurisdiction	Regulatory Pathway	Triggering Criteria	Example Product/Format	Translation Impact
USA (FDA)	361 HCT/P (under 21 CFR 1271)	Meets all 21 CFR 1271.10(a) criteria, including “minimal manipulation” and “homologous use.”	Intact or minimally manipulated amniotic/umbilical sheet allografts (e.g., AmnioGraft^®^, EpiFix^®^, EpiCord^®^).	No premarket approval required; tissue-cGTP and registration/listing apply.
USA (FDA)	Biologic/BLA (Section 351)	Fails one or more 361 criteria (e.g., undergoes “more-than-minimal manipulation” or is for “non-homologous use”).	Highly processed flowable/particulate matrices (e.g., Interfyl^®^, AmnioFill^®^) or placenta-derived MSC therapies currently in clinical trials.	Requires premarket review, extensive clinical evidence, and CMC requirements; lengthy and costly.
Europe (EMA)	Medical Device (under MDR)	Non-viable human tissue/cell-derived product intended for a medical-device purpose (classification depends on device rules).	Acellular structural scaffolds and commercialized dried amniotic membranes (e.g., Omnigen^®^).	Requires conformity assessment, clinical safety/performance evaluation, and CE marking via MDR routes.
Europe (EMA)	ATMP (Advanced Therapy Medicinal Product)	Contains viable cells/tissues and meets the ATMP definition (borderline cases reviewed by EMA/CAT).	Cultured placental MSC therapies or cell-laden bioprinted constructs (e.g., Pluristem’s PLX-PAD cells, currently in clinical development).	Subject to centralized EU framework; development is highly complex, restrictive, and costly.

## Data Availability

No new data were created or analyzed in this study. Data sharing is not applicable to this article.
